# Single-Atom Catalysts for Low-Temperature Thermocatalytic Ammonia Synthesis

**DOI:** 10.3390/molecules31081321

**Published:** 2026-04-17

**Authors:** Javier Arroyo-Caire, José María Abelleira-Pereira, Juan Carlos Serrano-Ruiz

**Affiliations:** 1 Materials and Sustainability Group, Department of Engineering, Universidad Loyola Andalucía, Avda. de las Universidades s/n, Dos Hermanas, 41704 Seville, Spain; jarroyo@uloyola.es; 2 Department of Chemical Engineering and Food Technology, Higher Technical School of Engineering of Algeciras (ETSIA), University of Cádiz, Algeciras, 11202 Cádiz, Spain; jose.abelleira@uca.es

**Keywords:** thermocatalysis, ammonia synthesis, single-atom catalysts, Ruthenium, mild conditions, nitrogen activation

## Abstract

Ammonia is indispensable to the fertilizer and chemical industries, yet its manufacture still relies predominantly on the energy-intensive Haber–Bosch process operated at 400–500 °C and 150–250 bar, with a substantial carbon footprint. Single-atom catalysts (SACs) and sub-nanometric clusters have recently emerged as promising alternatives for thermocatalytic ammonia synthesis under milder conditions because they maximize metal utilization and enable precise control of the active site environment. This review first summarizes how the transition from conventional Fe and Ru nanoparticles to isolated or few-atom sites fundamentally alters the kinetic landscape, favoring associative N_2_ activation pathways that lower apparent activation energies and alleviate H_2_ poisoning. We then discuss Ru-based SACs and SAAs supported on zeolites, carbons, ceria, and MXenes, highlighting how strong metal–support and promoter interactions, tandem single-atom/nanoparticle motifs, and alloying strategies tune N_2_ and H_2_ binding to deliver high NH_3_ productivities at 200–400 °C and ≤30 bar. In parallel, we review emerging non-noble systems based on Fe and Co, including high-loading Fe–N_4_ sites prepared via MOF-derived post-metal-replacement routes and Co single atoms or Co_2_ clusters on N-doped carbons, which already rival or surpass Ru benchmarks under similar conditions. Collectively, these studies show that tailoring the number of atom metal sites, coordination, and support polarity around isolated metal sites provides a useful tool to mitigate some aspects of volcano and scaling-relation limitations, indicating that SACs could contribute to low-temperature ammonia synthesis when combined with appropriate process design.

## 1. Introduction

Ammonia synthesis is one of the most important reactions in the chemical industry worldwide. Thus, the large-scale manufacture of nitrogen fertilizers relies almost exclusively on the thermocatalytic conversion of N_2_ and H_2_ via the Haber–Bosch (HB) process. While the HB process has enabled unprecedented growth of agricultural productivity, it consumes a substantial amount of energy, generating a significant carbon footprint. It is estimated that conventional HB plants consume a few percent of the global primary energy supply and contribute with a significant fraction of anthropogenic CO_2_ emissions [1], largely because H_2_ is still produced from fossil resources (e.g., natural gas via steam methane reforming, SMR) and because the process is operated at harsh temperature (400–500 °C) and pressure (150–250 bar) conditions to overcome kinetic barriers associated with cleavage of the highly stable N≡N bond. Also, ammonia is increasingly recognized to be a potential candidate as a carbon-free energy carrier owing to its high hydrogen content, high volumetric energy density relative to compressed H_2_, ease of liquefaction, and the possibility of direct utilization in fuel cells or combustion systems [2,3]. Thus, there is a large incentive to develop ammonia synthesis routes compatible with low-carbon electricity and decentralized production. In this sense, operating at lower temperatures is particularly attractive because it simultaneously allows higher equilibrium conversions while providing a new pathway to direct integration with intermittent renewable electricity sources for H_2_ generation and process heat management [4]. However, conventional Fe-based catalysts present very limited activity at temperatures below 400 °C as a result of their high activation energies (e.g., 150–200 kJ/mol). Mild-condition ammonia synthesis thus requires new catalysts that can activate N_2_ through alternative pathways with significantly lower activation energy, so that high turnover frequencies are maintained at moderate temperature (200–300 °C) and pressures (20–30 bar) [5,6]. These conditions are highly desirable since they (i) allow for better thermal coupling with solid-oxide or proton-exchange membrane electrolyzers; (ii) allow for the use of smaller, modular reactors with lower compression requirements; and (iii) reduce the capital and operational penalties associated with high-pressure and temperature operation.

The design of new catalysts able to operate under mild conditions is a challenging task that requires new materials with the ability to activate the very stable N_2_ molecule while keeping a good balance among the catalytic steps (i.e., adsorption, surface reaction and desorption). The difficulty of this challenge is summarized in the volcano relationship between the activity and the binding energy towards N_2_ [7,8]. The volcano plot encapsulates Sabatier’s principle and reveals that ammonia synthesis activity peaks at intermediate nitrogen-binding energies since overly strong adsorption slows N–H formation (surface reaction) and NH_3_ desorption, whereas too weak adsorption prevents efficient N_2_ activation. Additionally, the design of new catalysts is limited by the scaling relation dictated by Brønsted–Evans–Polanyi (BEP) [9]. According to this, the energetics of key steps of the ammonia synthesis reaction, such as N≡N dissociation and N–H bond formation, are linked to a single descriptor (e.g., N_2_ adsorption energy). Thus, selecting metals with proper N_2_ adsorption energy in an attempt to optimize the N_2_ activation step may result in NH_3_ desorption being negatively affected, an issue that becomes particularly important when targeting low reaction temperatures [10]. Traditional iron catalysts (e.g., magnetite-derived Fe phases promoted with alkali and alkaline earth oxides) operate via a dissociative mechanism in which N_2_ chemisorbs on and cleaves directly to multi-atom nanoparticles, followed by stepwise hydrogenation of adsorbed N to NH, NH_2_, and finally NH_3_. The optimal temperature–pressure window of the HB process is, therefore, dictated by the structural sensitivity of N_2_ dissociation at specific Fe sites [11]. Ru has emerged as a second-generation HB metal owing to its higher intrinsic activity at lower temperatures [12]. Industrial adoption, however, has been very limited due to Ru scarcity, cost, the susceptibility of carbon supports to undergo methanation, and the need for high Ru loadings and complex promoters. Both Fe and Ru catalysts remain limited by hydrogen poisoning (a classical issue of Ru) and by BEP correlations.

Single-metal catalysts (SACs) and single-atom alloys (SAAs) have been recently proposed as promising, but still emerging, materials for mild-temperature HB processes [13,14]. In SACs, atomically dispersed metal centers are coordinated with ligand environments, which provides stability and allows the metal electronic structure to be finely tuned via local ligand field and metal–support interactions. Also, SACs provide maximum metal utilization while homogenizing active sites. In the context of ammonia synthesis, SACs provide an alternative mechanistic approach by favoring associative pathways in which N_2_ is partially hydrogenated prior to N–N bond cleavage, thereby circumventing some of the scaling-relation constraints of nanoparticle-based Fe and Ru catalysts [15,16]. At the same time, SACs for thermocatalytic ammonia synthesis remain an emerging class, and issues such as limited operating lifetimes, sensitivity to support reconstruction, challenges in scaling up synthetic routes, and the still modest number of systems tested under truly industrially relevant conditions highlight that their practical deployment is far from established. Despite these advances, the existing literature on this topic still lacks a focused, mechanism-oriented review of single-atom and closely related catalysts for thermocatalytic ammonia synthesis under genuinely mild Haber–Bosch conditions. Recent reviews either concentrate on Ru single-atom catalysts across different modalities or on Ru-embedded systems without a systematic cross-comparison with earth-abundant metals (e.g., Ru SACs for NH_3_ synthesis and related processes [17]) or treat single-atom, sub-nanometric clusters, and larger ensembles together with an emphasis on structural classification rather than on kinetic descriptors (e.g., [13]). In contrast, this review specifically targets thermocatalytic NH_3_ synthesis in the 200–400 °C and ≤30 bar window and proposes a unified framework to compare Ru-based and non-noble SACs, SAAs, and atomic clusters on the basis of activity, apparent activation energy, reaction orders, and experimental signatures of associative versus dissociative pathways while explicitly linking these kinetic features to metal nuclearity, coordination environment, and support polarity. With this in mind, this review critically covers the current progress in SACs for thermocatalytic NH_3_ synthesis under mild conditions, with particular emphasis on how their tailored coordination environments and cooperative active sites can be exploited to overcome volcano and BEP limitations, and to guide the rational design of next-generation HB catalysts.

Beyond thermocatalysis, recent theoretical studies have also proposed single-superatom motifs such as TiO, ZrO, and WC supported on graphene as promising catalysts for the electrochemical nitrogen reduction reaction, highlighting that atomically precise catalyst design may extend beyond isolated metal atoms toward cluster-like motifs with tailored electronic structure [18]. Although these systems fall outside the thermocatalytic scope of the present review, they illustrate the breadth of emerging concepts in ammonia-related catalysis. More broadly, ligand field engineering in atomically dispersed sites remains a powerful lever in ammonia-related catalysis, as also illustrated by theoretical studies on coordinated macrocyclic motifs for electrocatalytic N_2_ reduction, including Mo-, Re-, and Tc-containing phthalocyanine-like systems [19]. Although these reports fall outside the thermocatalytic focus of this review, they reinforce the importance of metal coordination chemistry in tuning N_2_ activation energetics.

## 2. Reaction Mechanisms and Scaling in the Thermocatalytic Synthesis of NH_3_

**Figure 1:**
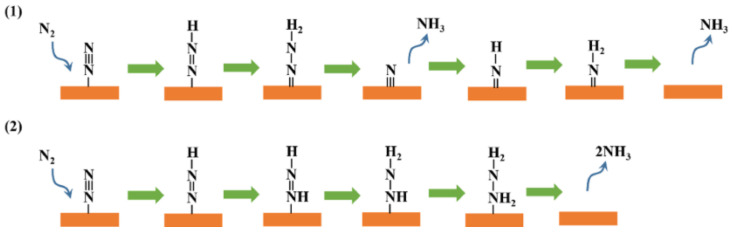
Schematic representation of distal (**1**) and alternate (**2**) associative pathways. Adapted from [22], licensed under CC BY-NC-ND 4.0.

Classical thermocatalytic ammonia synthesis over Fe and Ru nanoparticles takes place predominantly via a dissociative pathway in which N_2_ chemisorbs and cleaves directly on multi-atom sites right before sequential hydrogenation to NH, NH_2_, and finally NH_3_ occurs. N_2_ cleavage usually takes place on stepped Fe and Ru surfaces (e.g., Fe C_7_ and Ru B_5_ ensembles) in a step that requires significant energy and results in high apparent activation energies for the reaction. Catalysts promoting this classical mechanism are highly limited by the volcano relationship between the activity and the N_2_ binding energy, the so-called scaling relation [10]. Alternatively, N_2_ can undergo partial hydrogenation before N-N cleavage in an associative mechanism well known in homogeneous and electrocatalytic ammonia synthesis routes [20,21]. Very recently, this mechanism has been proposed for thermocatalytic ammonia synthesis [13,22]. As shown in Figure 1, the associative mechanism can present two potential pathways, namely, distal and alternating pathways. In the distal pathway, one N atom in adsorbed N_2_ is progressively hydrogenated to form NNH, NNH_2_, and NNH_3_ intermediate species, with N–N scission occurring at a late stage. In the alternate pathway, both N atoms are hydrogenated consecutively to form intermediates such as NHNH and NH_2_NH_2_. The main signatures of this mechanism are as follows: (i) ultralow activation energies in the range of 20–30 kJ/mol, indicative of a mechanism in which N_2_ breaking is not taking place; (ii) the highest energy barrier is shifted from bare N_2_ dissociation to hydrogenation of N_2_ or N_2_H_x_ species; (iii) intermediate species such as early N–N–H species (e.g., N_2_H, NNH, and NNH_2_, proposed by DFT [23]), N_2_H_2_ and N_2_H_3_ (detected by TOF-MS [13]); and hydrazine (N_2_H_4_), which can be detected by IR and UV techniques [22]. The main advantage of the associative mechanism is that it can weaken the strict BEP linkage between N_2_ activation and NH_3_ desorption that constrains conventional Fe and Ru catalysts, thereby providing new possibilities in catalyst design, particularly useful for low-temperature applications, although this has so far been demonstrated mainly in theoretical studies and a limited number of experimental systems.

Among these new catalyst designs, SACs have been recently proposed as outstanding materials to carry out ammonia synthesis at low temperatures. Thus, on isolated sites, multi-atom configurations similar to the Fe C_7_ or Ru B_5_ ensembles are lacking, such that the classical direct dissociation mechanism is strongly disfavored both geometrically and electronically. In fact, combined theoretical and experimental studies have shown that Ru [24] and Fe [25] SACs support a picture in which N_2_ binds in end-on or slightly bent geometries and is likely converted into N_2_H or N_2_H_2_ prior to N–N scission, consistent with the substantially lower apparent activation energies measured relative to nanoparticle counterparts, although the full reaction coordinate is still primarily inferred from DFT. This shift in mechanism is particularly important under mild temperature (200–350 °C) and pressure (≤30 bar) conditions, where dissociative pathways become kinetically sluggish, whereas associative routes can provide significant NH_3_ synthesis rates.

Apparent activation energies and reaction orders for ammonia synthesis change markedly when using SACs and sub-nanometric clusters, in line with a shift of the rate-determining step toward associative N-hydrogenation steps or NH_3_ desorption in many systems; however, in most cases, this assignment remains indirect and rests on trends in kinetics combined with DFT rather than on direct observation of every intermediate. Thus, when ammonia synthesis catalysts transition from conventional nanoparticles to single atoms and sub-nanometric clusters, the apparent activation energy typically decreases to ultralow values and, as indicated above, the rate-determining step shifts to associative N_2_ hydrogenation steps or NH_3_ desorption. In parallel, the kinetic orders evolve from strongly positive in N_2_ and strongly negative in H_2_ to lower positive N_2_ orders and weakly positive or near-zero H_2_ orders, indicating that N_2_ activation becomes easier and H_2_ poisoning is substantially alleviated. This issue is particularly critical when operating at low temperatures, where hydrogen adsorption is thermodynamically favored and can limit catalyst productivity. On the other hand, recent studies report positive N_2_ and H_2_ reaction orders, above the unity, for catalysts driven by the associative mechanism, which can be ascribed to the competition of N* and H* species to occupy the surface-active sites, resulting in an excellent pressure effect [26,27]. While many reports invoke associative pathways for SACs, rigorous experimental confirmation is still rare and often relies on indirect kinetic trends; we, therefore, highlight below (Section 5) those systems where direct or quasi-direct evidence exists.

The mechanistic and kinetic aspects discussed above indicate that SACs and SAAs are well-suited candidates for exploiting the associative N_2_ activation to partially alleviate the scaling and H_2_-poisoning constraints that limit classical Fe and Ru catalysts. As will be discussed in the following sections, by tailoring the local coordination, electronic structure, and metal–support (or metal–metal) interactions around the metal center, these catalysts can simultaneously lower the barrier for N_2_H_x_ formation, moderate the binding of N and NH_x_ species, and provide sustained high turnovers under H_2_-rich, low-temperature conditions.

## 3. Ru-Based SACs and SAAs

The high activity of Ru NPs in this reaction has encouraged researchers to investigate the kinetic behavior of Ru isolated atoms to move the thermocatalytic ammonia synthesis toward lower temperatures and pressures. Thus, Ru-based SACs and SAAs have been proposed for low-temperature ammonia synthesis applications (Table 1). As shown in Table 1, low metal loadings are typically used for this application. Higher loadings typically lead to the onset of higher Ru ensembles or even NPs [28,29], which coexist with single atomic sites. The support plays a crucial role in enabling and maintaining maximum metal dispersion. For example, the use of zeolites and related materials (e.g., HZSM5) with well-defined zeolite cages favors confinement of metal entities, resulting in maximum metal dispersions at very low metal loadings (e.g., 0.2 wt%) [24,30]. On the other hand, the use of N-doped carbonaceous supports favors metal dispersion, although higher metal loadings are necessary to obtain synthesis rates in the range of a few mmol/g h [31]. Carbon materials are by far the most widely used supports for SACs and SAAs owing to their high surface area, tunable N-doping, good electronic conductivity, and ability to stabilize isolated metal sites [32]. However, under typical thermocatalytic ammonia synthesis conditions (e.g., 400 °C and moderate H_2_/N_2_ pressure), carbon supports can undergo methanation, leading to CH_4_ formation and potential catalyst deactivation via support loss and Ru sintering. A careful monitoring of the outlet gas composition is, therefore, required to rule out CH_4_ formation. This represents a major practical obstacle for long-term operation of Ru/C-type SACs under H-rich, high-temperature Haber–Bosch conditions. As emphasized by Zhou et al. [29] and Zhang et al. [31], highly graphitized or otherwise stabilized carbons are suggested to suppress this side reaction.

**Figure 2:**
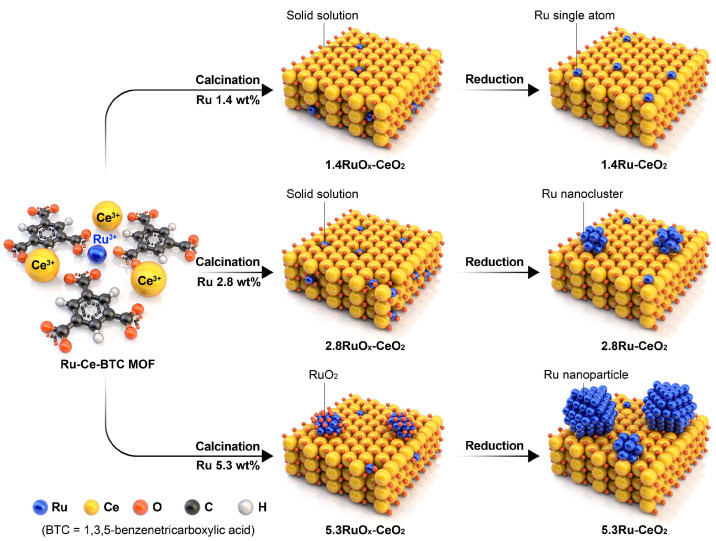
Schematic illustration of the MOF-derived Ru–Ce–BTC route to Ru/CeO_2_ catalysts, showing how varying Ru loading (1.4, 2.8, and 5.3 wt%) leads after calcination and reduction to Ce_1−x_Ru_x_O_2_ solid solutions with isolated Ru single atoms (1.4Ru–CeO_2_), surface Ru nanoclusters (2.8Ru–CeO_2_), or RuO_2_-derived Ru NPs (5.3Ru–CeO_2_). Reproduced from [28] with permission under the Creative Commons Attribution 4.0 International (CC BY 4.0) license.

Apart from zeolites and carbon materials, active supports such as reducible oxides (e.g., CeO_2_) have been used to promote the performance of Ru in the ammonia synthesis reaction [33]. CeO_2_ has been found to promote Ru mainly by providing oxygen vacancies and increasing electron donation to Ru via strong metal support interactions. As a result, the barrier for N≡N dissociation is lowered via electron donation towards the antibonding molecular bonds of the N_2_ molecule. Additionally, hydrogen poisoning on Ru is alleviated by hydrogen spillover over Ru-Ce^3+^ interfacial sites, which is kinetically reflected on positive reaction orders for H_2_ and lower activation energies and higher NH_3_ synthesis rates at lower temperatures as compared to Ru catalysts on regular unreducible oxides [34,35]. These promoting effects of CeO_2_ on Ru were exploited for SACs by Sivan et al. [28] MOF-derived one-pot strategy to generate Ru SACs, nanoclusters, and nanoparticles on CeO_2_ supports (Figure 2). The SAC-rich sample contained predominantly isolated Ru atoms incorporated into a Ce_1−x_Ru_x_O_2_ solid solution and showed superior activity and lower activation energy as compared to a conventional NP-based Ru/CeO_2_ catalyst. In addition, reaction-order analysis revealed hydrogen poisoning to be largely suppressed on both SACs and nanocluster-based Ru catalysts. The optimized nanocluster catalyst showed moderate N_2_ and positive H_2_ reaction orders, consistent with a mixed associative/dissociative mechanism in which Ru nanoclusters on oxygen-vacancy-rich CeO_2_ patches promote classical N_2_ dissociation, while nearby cationic Ru–Ce sites participate in sequential associative N_2_H_x_ formation. Long-term tests on the optimized nanocluster catalyst showed stable activity with no significant sintering or phase change after reaction, while using XRD and XAFS on post-reaction samples confirmed that both the SAC and nanocluster structures remained largely preserved after short-term reactions. In their catalytic tests, the 2.8 wt% Ru–CeO_2_ nanocluster catalyst displayed stable NH_3_ formation rates over the investigated time-on-stream at 400–425 °C and 10–30 bar, with no signatures of hydrogen poisoning relative to Ru nanoparticles. Post-reaction XRD patterns did not show additional reflections attributable to metallic Ru, and XAFS analysis indicated that Ru remained highly dispersed and strongly interacted with the CeO_2_ lattice. Together with electron-microscopy evidence for the coexistence of isolated Ru sites and sub-nanometric clusters after reaction, these observations suggest that the MOF-derived one-pot synthesis provides a sufficiently robust anchoring environment to limit Ru aggregation under the short-term thermocatalytic conditions examined in this study. Zhou et al. [29] continued this approach by engineering a Ru single-atom/nanoparticle tandem catalyst (Ru_1_ + NPCe_2_/GC), where Ru_1_ sites and Ru NPs co-existed, anchored to CeO_2_ nano-islands supported on graphitized carbon. An outstanding NH_3_ synthesis rate of 59 mmol g ^−1^ h^−1^ at 400 °C and 1 MPa and an apparent activation energy as low as 56 kJ mol^−1^ were achieved with a Ru loading of ca. 3.5 wt%. Kinetic analysis revealed reduced N_2_ reaction orders and positive H_2_ reaction orders as compared to a ceria-free NP-based counterpart, which emphasizes the crucial role of the reducible oxide in improving the kinetic signatures of Ru. Hydrogen spillover from Ru to adjacent Ru_1_–CeO_2_ sites was again raised to explain suppression of hydrogen poisoning. A dual associative–dissociative pathway, with RuNPs responsible for dissociative N_2_ activation and Ru_1_ sites operating via an associative mechanism, was corroborated by isotope-exchange and TOF-SIMS experiments. Importantly, Ru_1_ + NPCe_2_/GC exhibited excellent stability under Haber–Bosch conditions. Thus, in fixed-bed tests at 400 °C and 1 MPa, the catalyst sustained an NH_3_ synthesis rate of 59.0 mmol g^−1^ h^−1^ for at least 600 h without detectable deactivation, and a 550 g scaled-up, molded K–Ru_1_ + NPCe_2_/GC bed delivered a nearly constant mass-specific rate of ca. 1870 mmol g^−1^ h^−1^ over an additional 600 h, outperforming a commercial Ru catalyst under identical conditions. Post-reaction AC-HAADF-STEM, XAFS and XANES analyses showed that the valence state and Ru–O/Ce coordination environment closely resembled those of the fresh material and that Ru single atoms and nanoparticles remained co-localized on CeO_2_ nano-islands without obvious growth of large Ru aggregates, confirming that the tandem Ru_1_/RuNP architecture and the high-crystallinity graphitized-carbon support are structurally robust and resistant to Ru sintering, support methanation, or phase separation for over 1000 h of continuous operation.

**Table 1:** Catalytic performance of Ru-based SACs and SAAs in the NH_3_ synthesis.

Catalyst	Metal Loading (wt%)	Reaction Conditions (°C/bar/mL g^−1^ h^−1^)	NH_3_ Rate (mmol/g h)	Ea (kJ/mol)	R Orders (α, β, γ)	Stability	Ref.
Ru/CeO_2_	1.4	400; 10; 72,000	4	72	0.62, 0.27, −0.39	Not provided for SACs. Nanoclusters showed stable activity for 200 h on stream, with slight decrease in metal dispersion after reaction.	[28]
Ru/CeO_2_/graphite SACs and NPs	3–3.5	400; 10; 60,000	59	56–101	0.4–0.8; 0.8–0.2; –	Stable for 1100 h; Ru_1_/RuNP preserved structure and negligible CH_4_ formation.	[29]
Ru/SiO_2_ zeolite	0.27	360; 1; 18,000	4.7	55	0.15; 0.36; –	Slight deactivation after 100 h on stream. Ru remained as isolated single atoms in the zeolite framework after reaction, as confirmed by STEM, XAS, and in situ DRIFTS.	[24]
Ru/HZSM5	0.20	400; 10; 60,000	9	38	0.86; 0.88; –	Ru/HZ SAC maintains a constant NH_3_ synthesis rate over 78 h at 400 °C, with post-reaction AC-STEM and XRD confirming only isolated Ru single atoms and no bulk Ru phases.	[30]
Ru-Fe/LDHs	0.1	400; 10–50; 10,000	3–10	—	—	Significant initial activity decrease for first 40 h on stream (Ru sintering and H_2_ poisoning) followed by stabilization. Stability test carried out at 400 °C and 50 bar. Structure preserved.	[36]
RuCo/N-doped C	0.83–2.45	400; 30; 60,000	3–10	76–117	0.41; 0.55; −0.51	Ru_1.7_Co_1_ maintained NH_3_ synthesis rate for 100 h at 400 °C and 3 MPa with negligible CH_4_ formation. Ru–Co alloy and carbon structure preserved (STEM, EDS, XRD, Raman).	[31]

The performance of Ru in the ammonia synthesis reaction can also be improved by dispersing it in highly confined environments such as those provided by zeolites. With a very low Ru loading (ca. 0.27 wt%), Qiu et al. [24] prepared Ru SACs with synthesis rates comparable to those of a conventional Cs-promoted Ru/MgO catalyst. Kinetic analysis revealed a low apparent activation energy of 55 kJ mol^−1^ and unusually low N_2_ and positive H_2_ reaction orders (α ≈ 0.15, β ≈ 0.36). XANES/EXAFS revealed Ru to be in a high oxidation state close to +4 in four-fold coordination with the zeolite framework. DFT calculations on the four-coordinate Ru single-atom site revealed N_2_ to adsorb molecularly, with N≡N cleavage taking place only after hydrogenation by H_2_ species located in the zeolite channel, which, along with the kinetic data, supported the associative route. Stability tests showed that Ru SAs/S-1 produced ammonia at an essentially constant rate over 100 h at 0.1 MPa, with no detectable loss of activity, and even under more demanding conditions (1.0 MPa), SAC outperformed Cs–Ru/MgO benchmark catalysts that started to deactivate after ca. 30 h on stream. Post-reaction AC-HAADF-STEM and Ru K-edge XANES/EXAFS revealed nearly identical spectra and atomically dispersed Ru environments before and after reaction, with a single dominant Ru–O shell (coordination number of ca. 4, Ru–O of ca. 2.01 Å) and no Ru–Ru contributions, confirming that the oxidized Ru centers remained as isolated atoms strongly anchored to the S-1 framework. Complementary operando CO-DRIFTS showed that the characteristic mono-, di-, and tricarbonyl bands associated with cationic Ru^δ+^ sites were fully recovered after 3 h under NH_3_ synthesis conditions at 633 K, with only noise-level bridging-CO features, further demonstrating the absence of Ru aggregation and highlighting the robustness of the zeolite cages in preventing single-atom sintering under strongly reducing, high-temperature conditions. Wang et al. [30] also exploited the channeled structure of ZSM5 to prepare Ru SACs with low metal loadings. NH_3_ synthesis rates of ca. 9 mmol g^−1^ h^−1^ were achieved at 400 °C, 10 bar, and 60,000 mL g^−1^ h^−1^, clearly surpassing conventional Ru nanoparticle catalysts under comparable conditions while using substantially less Ru. Remarkably, a very low apparent activation energy (ca. 38 kJ mol^−1^) and kinetic parameters (α ≈ 0.86; β ≈ 0.88 with a moderately negative NH_3_ order) pointed to an associative pathway where N_2_ activation is facilitated and hydrogen does not poison the cationic, highly oxidized Ru centers anchored to the aluminosilicate framework. A thermal stability test at 400 °C and 10 bar showed that Ru/H-ZSM-5 maintained an essentially constant NH_3_ synthesis rate over 78 h on stream, with only a slight initial decline before reaching a steady state, indicating that no progressive deactivation occurs under these mild Haber–Bosch conditions. Post-reaction AC-STEM imaging of the used catalyst revealed exclusively isolated Ru single atoms in the ZSM-5 channels, with no discernible Ru clusters or nanoparticles, while XRD patterns showed no peaks attributable to bulk Ru phases, together confirming that the microporous aluminosilicate framework effectively prevents Ru aggregation during operation. In addition, the NH_3_ rate increased roughly linearly with pressure up to 5 MPa without signs of hydrogen poisoning, and kinetic parameters remained unchanged within error after the stability test, suggesting that the local electronic structure of the cationic Ru centers is preserved and that the zeolite confinement provides a robust environment against both sintering and H_2_-induced deactivation over the tested timescale.

Apart from tailoring the local environment of isolated Ru atoms within zeolite frameworks, an alternative strategy to boost Ru performance in ammonia synthesis is to alloy it with a second metal, creating SAAs in which both electronic and geometric effects allow for the fine-tuning of N_2_ and H_2_ activation at the Ru site. Singh et al. [36] developed robust Ru–Fe SAA catalysts on MgFeO_x_ layered double hydroxides (LDHs). The optimal catalyst (with only 0.1 wt% Ru) showed activities rivaling or surpassing those of conventional Ru catalysts with orders-of-magnitude more Ru loadings. DFT and advanced spectroscopy revealed that isolated Ru atoms incorporated into Fe–Ru alloy particles weakened the adsorption of both molecular and atomic nitrogen on Fe (110), thereby facilitating intermediate desorption and accelerating the overall reaction without severe H_2_ poisoning. A 150 h durability test at 400 °C and 5 MPa showed that the optimized MgFeO_x_-0.1Ru SAA catalyst maintained a nearly constant NH_3_ synthesis rate of ca. 7217 µmol g^−1^ h^−1^, with no progressive loss of activity over the entire run, evidencing excellent stability under industrially relevant conditions. Post-catalytic XRD patterns confirmed that the MgFeO_x_ support preserved its crystalline structure and did not generate new Ru-containing bulk phases, while high-resolution TEM and synchrotron EXAFS/Mössbauer analyses showed that Ru remained atomically dispersed within small (ca. 1.6 nm) Fe–Ru alloy particles, without the appearance of large Ru aggregates, indicating that the SAA configuration and LDH-derived oxide matrix effectively suppress Ru sintering during long-term operation. The addition of Co to Ru also resulted in significant changes in the kinetic behavior of Ru in the ammonia synthesis reaction [31]. Apparent activation energies of 76 kJ mol^−1^ were found for Ru_1.7_Co_1_, substantially lower than the 117 kJ mol^−1^ measured for Ru_2_Co_1_, aligning with the observed activity trend and revealing an optimal Ru/Co ratio for N_2_ activation. The reaction orders of Ru_1.7_Co_1_ SAA (α = 0.41 and β = 0.55) contrasted with those of Ru_2_Co_1_ (α = 1.10, β = 1.28) and conventional Ru catalysts. Long-term tests at 400 °C and 10 MPa showed that Ru_1.7_Co_1_ SAA maintained a nearly constant NH_3_ synthesis rate over 100 h on stream, with no measurable loss of activity, whereas Ru_2_Co_1_, despite its higher Ru content, did not exhibit any stability advantage, underscoring that simply increasing Ru loading cannot compensate for loss of the optimal SAA structure. Post-reaction XRD and AC-HAADF-STEM/EDS confirmed that the Ru–Co alloy nanoparticles and N-doped carbon support retained their morphology, and XAS/XPS analyses revealed that Co remained atomically dispersed and strongly coordinated to Ru (Co–N and Ru–Co features preserved, with no Co–Co or segregated Ru signatures), indicating that the electronic coupling between Ru and Co and the single-atom-alloy motif are structurally robust under prolonged operation without detectable Ru or Co segregation.

Ru SACs supported on MXenes such as Mo_2_CO_x_ have been theoretically proposed to have the potential to promote thermocatalytic NH_3_ synthesis at low temperatures [37]. In this model system, a single Ru atom is anchored at an oxygen vacancy of defective Mo_2_CO_x_, forming a thermodynamically stable configuration that transfers up to 0.87 e to adsorbed N_2_, leading to strong activation of the N≡N bond. Microkinetic analysis identified an associative pathway as the most favorable route, with the rate-determining step being the *NH_2_ → *NH_3_ step. The Mo_2_CO_x_ substrate was described to serve as an electron reservoir that donates charge to NHx intermediates, while the Ru single atom serves as a bridge for charge transfer between the MXenes and the adsorbates. Because these conclusions are drawn from atomistic modeling without experimental validation so far, they should be regarded as hypotheses that motivate synthesis and operando testing of Ru/MXene SACs rather than as established mechanistic facts.

## 4. Non-Noble SACs

**Figure 3:**
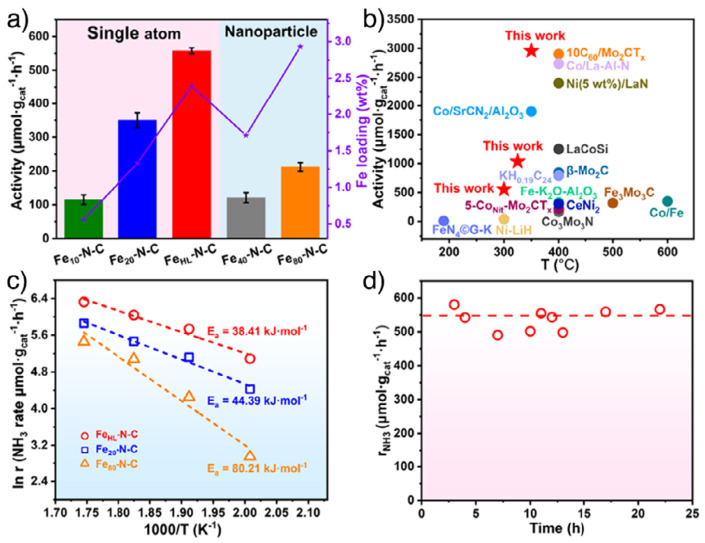
Catalytic performance of high-loading Fe single-atom catalysts under mild conditions. (**a**) NH_3_ synthesis rates of Fe_x–N–C samples and FeHL–N–C measured at 300 °C and 0.1 MPa under a WHSV of 30 L g_cat^−1^ h^−1^ with a 25% N_2_/75% H_2_ feed; error bars denote the standard deviation from three independent runs. (**b**) Benchmarking of FeHL–N–C against previously reported non-noble metal catalysts for NH_3_ synthesis at atmospheric pressure, highlighting its superior productivity in the low-temperature regime. (**c**) Arrhenius plots for FeHL–N–C and Fe_x_–N–C (x = 20, 40, 80), illustrating that FeHL–N–C retains low apparent activation energies despite its higher Fe loading. (**d**) Time-on-stream stability of FeHL–N–C at 300 °C and 0.1 MPa, showing essentially constant NH_3_ rate over the tested period. Reproduced with permission [25]. Copyright © 2025 Wiley-VCH GmbH.

Selected earth-abundant metal SACs and clusters have been reported with competitive NH_3_ productivities under specific, often idealized, conditions compared to Ru benchmarks, suggesting a potential route to alleviate cost and supply constraints, though broader validation is still required. Table 2 compiles representative non-noble metal-based SACs reported for low-temperature thermocatalytic ammonia synthesis. Early efforts on non-noble SACs focused on Fe single sites supported on N-doped carbon. In this sense, Jiang et al. [25] prepared Fe/N-doped carbon with atomically dispersed Fe–N_4_ motifs at noticeably high Fe loadings (2.39 wt%). These high loadings were achieved by using a MOF-derived post-metal-replacement strategy based on porphyrinic Fe_x-PCN-222. Pyrolysis yielded Zn_1_–Fe_1_–N–C/ZrO_2_ with isolated Fe–N_4_ and Zn–N_4_ sites, which acid etching converts into Zn-vacancy-rich Znv–Fe_1_–N–C that retained single-atom Fe but introduces coordinatively unsaturated sites. Refilling these vacancies with additional Fe atoms yielded the final material, which, as confirmed by EXAFS and XPS, consisted exclusively of Fe–N_4_ motifs and no Fe–Fe scattering. FeHL–N–C reached NH_3_ synthesis rates of 558 µmol g_cat^−1^ h^−1^ at 300 °C and 1 bar, outperforming both fused-iron benchmarks and Ru-based reference catalysts under similar conditions and placing this system among the most active non-noble SACs reported to date (Figure 3a,b). Apparent activation energies in the range of 38–44 kJ mol^−1^ (Figure 3c) and the weak sensitivity of the rate to total pressure between 1 and 50 bar revealed an associative-type mechanism in which N_2_ activation was significantly facilitated relative to classical Fe nanoparticles. Time-on-stream tests at 300 °C and 0.1 MPa (Figure 3d) showed that FeHL–N–C sustained its NH_3_ production rate for more than 20–25 h without significant loss of activity, and the rate increased approximately linearly with total pressure up to 5 MPa, indicating that hydrogen poisoning is effectively avoided on the single-atom Fe–N_4_ sites. Isothermal H_2_-reduction experiments at 300 °C and 0.1 MPa detected no CH_4_ formation, demonstrating the high stability of the N-doped carbon support under strongly reducing conditions, while post-reaction XRD and TEM/HAADF-STEM revealed no new Fe-containing crystalline phases or Fe–Fe aggregates, fully consistent with the persistence of atomically dispersed Fe and a structurally intact Fe–N_4_ framework after reaction. This work is particularly relevant in that it provides a robust methodology to prepare rarely achieved, highly loaded single-atom Fe catalysts whose activities under mild conditions are comparable to those of precious metals such as Ru. Chen and co-workers [38] confined Fe single atoms within a N- and K-doped graphene lattice to further tune the electronic environment around Fe and stabilize isolated active sites. Despite the relatively low NH_3_ synthesis rate (10^−3^–10^−2^ mmol g^−1^ h^−1^ at 150–190 °C and 1 bar) and a high apparent activation energy of ca. 92 kJ mol^−1^, the catalyst exhibited notable structural robustness: time-on-stream tests for ca. 30 h at near-ambient pressure with essentially unchanged NH_3_ productivity. Post-reaction TEM/HAADF-STEM and XRD detected only atomically dispersed Fe with no nanocluster or metallic Fe reflections, indicating that the Fe–N,K–graphene framework effectively suppresses Fe migration and aggregation under these mild, H_2_-rich conditions. Together, these two Fe systems illustrate how increasing ligand field strength (from disordered N-doped carbon to well-defined graphitic coordination) allow to operate at lower temperature and pressure while keeping excellent structural stability.

Cobalt SACs supported on N-doped carbons have recently emerged as particularly promising non-noble catalysts, offering higher intrinsic activities while still exploiting associative-type pathways. Wang et al. [39] reported Co single atoms stabilized on N-doped hollow carbon spheres (3.73 wt% Co) that reached 4.3 mmol g^−1^ h^−1^ at 350 °C, 10 bar, and 30,000 mL g^−1^ h^−1^, with an apparent activation energy of ca. 50 kJ mol^−1^. Importantly, under 350 °C, 10 bar and 30,000 mL g^−1^ h^−1^, the Co–N–C catalyst maintained a stable NH_3_ synthesis rate over at least 100 h on stream, indicating that no progressive deactivation occurred despite the relatively low operating pressure and the coexistence of dynamic and steady-state Co sites. Post-reaction Co K-edge XAS and EXAFS showed virtually unchanged spectra compared to the fresh material, with Co present as isolated Co_1_–N_3.5_/Co–N_x_ moieties and no discernible Co–Co coordination, while HAADF-STEM confirmed the preservation of atomically dispersed Co centers on the N-doped hollow carbon shells and the absence of Co nanoparticles, demonstrating that the defect-rich carbon scaffold and pyrrolic/pyridinic N anchors provide a robust coordination environment that immobilizes single Co atoms and prevents their aggregation under prolonged high-temperature, high-pressure Haber–Bosch conditions. In kinetic terms, these authors confirmed that downsizing Co to single atoms favored associative N_2_ activation, as reflected in lowered activation barriers relative to classical Co/MgO or Co nitride catalysts. Peng et al. [40] extended this idea by modulating the local Co–N coordination number on porous N-doped carbon spheres, thus providing a direct link between coordination environment and the kinetic results. Their Co–N_2_ catalyst (3.20 wt% Co) exhibited an NH_3_ rate of 2.7 mmol g^−1^ h^−1^ at 300 °C and 10 bar alongside reduced N_2_ and moderately positive H_2_ reaction orders (α ≈ 0.45; β ≈ 0.27), which revealed facile N_2_ activation and alleviated hydrogen poisoning compared to nanoparticulate Co. A 50 h durability test at 300 °C and 10 bar showed that the Co–N_2_ catalyst maintained an essentially constant NH_3_ synthesis rate, with no measurable deactivation, indicating that the tailored Co–N_2_ coordination environment remains intact under continuous operation. Post-reaction characterizations (XAS and aberration-corrected STEM) revealed nearly unchanged Co K-edge spectra and local coordination parameters compared to the fresh sample, together with the absence of Co–Co scattering or metallic Co features and no detectable growth of Co nanoparticles, confirming that the porous N-doped carbon spheres provide a robust support that stabilizes Co atomic dimers/clusters and prevents further aggregation under the applied reaction conditions. When viewed together with the hollow-sphere Co SACs, this study underscores how fine-tuning Co coordination can be used to balance N_2_ binding, hydrogenation kinetics, and desorption steps to optimize performance at modest temperature conditions.

Finally, Zhou et al. [41] pushed non-noble systems into the sub-nanometric regime by synthesizing Co_2_ atomic clusters (Co_2_ ACCs) on N-doped carbon. Despite an ultralow Co loading of 0.90 wt%, Co_2_ ACCs achieved 8.5 mmol g^−1^ h^−1^ at 400 °C and 10 bar, with an apparent activation energy as low as 49 kJ mol^−1^ and kinetic orders of α = 0.48 and β = 0.72. These values revealed a further shift toward an associative mechanism in which N_2_ hydrogenation steps become rate-controlling, while N≡N cleavage is no longer the dominant barrier, consistent with extensive in situ spectroscopy revealing strong metal–support and inter-cluster interactions that promote electron donation from Co d-orbitals to the antibonding orbitals of N_2_. Long-term durability measurements under relevant conditions (350 °C, 1 MPa) showed that Co_2_ ACCs maintained an essentially constant NH_3_ synthesis rate over 51 h on stream, with no measurable deactivation and only trace CH_4_ signals in the outlet, indicating that neither Co sintering nor carbon hydrogenation proceeds to an extent that impacts performance over this timescale. Post-reaction powder XRD patterns were virtually identical to those of the fresh catalyst, displaying only the (002) reflection of graphitic carbon and no new peaks assignable to metallic Co, while HAADF-STEM and EXAFS analyses continued to show well-defined, sub-nanometric Co_2_ clusters homogeneously dispersed on the N-doped carbon and an absence of larger Co particles, demonstrating that the strong metal–support interaction and optimized Co–N coordination efficiently maintained the Co_2_ motif in place and preserve both cluster structure and carbon scaffold under prolonged high-temperature operation.

To facilitate a more quantitative comparison with state-of-the-art practice, Table 3 summarizes representative parameters for conventional Haber–Bosch technology, including typical operating windows (temperature, pressure, and space velocity), catalyst formulations currently used in large-scale ammonia plants, and characteristic NH_3_ productivities under industrial conditions. This benchmark provides a practical reference frame against which the mild-condition single-atom and cluster catalysts compiled in Table 1 and Table 2 can be interpreted.

**Table 2:** Catalytic performance of non-noble-based SACs in the NH_3_ synthesis.

Catalyst	Metal Loading (wt%)	Reaction Conditions (°C/bar/mL g^−1^ h^−1^)	NH_3_ Rate (mmol/g h)	Ea (kJ/mol)	R Orders (α, β, γ)	Stability	Ref.
Fe/N-doped C	2.39	300; 1; 30,000	0.1–0.5	38–44	Not measured	Fe SACs showed stable dispersion with no Fe agglomeration or methane formation for 25 h on stream as revealed by XRD and TEM. No H_2_ poisoning over 1–50 bar.	[25]
Fe/N and K-doped graphene	2.1	150–190; 1; 30,000	0.001–0.01	92	Not measured	Stable NH_3_ synthesis over 30 h at 170 °C and 1 bar; Fe remained as isolated single sites (no aggregation) according to post-reaction characterization.	[38]
Co/N-doped hollow carbon spheres	3.73	350; 10; 30,000	4.3	50	Not measured	Constant NH_3_ synthesis rate over 100 h on stream at 350 °C and 1 MPa, with Co remaining atomically dispersed (no Co–Co coordination or nanoparticle formation by XAS/HAADF-STEM).	[39]
Co/N-doped porous carbon spheres	3.20	300; 10; 60,000	2.7	—	0.45; 0.27; –	Stability test for 50 h on Co–N_2_ at 300 °C and 1 MPa with essentially constant NH_3_ rate.	[41]
Co_2_/N-doped carbon	0.90	400; 10; 60,000	8.5	49	0.48; 0.72; –	Co_2_ ACCs showed essentially constant NH_3_ synthesis activity for 51 h at 350 °C and 1 MPa with no detectable CH_4_ formation, HAADF-STEM and XRD confirmed that the Co_2_ cluster structure and carbon support remained unchanged under reaction conditions.	[40]

**Table 3:** Representative parameters for conventional Haber–Bosch ammonia synthesis.

Process	Catalyst	Reaction Conditions (°C/bar/mL g^−1^ h^−1^)	NH_3_ Rate (mmol/g h)	Ea (kJ/mol)	Summary of Operating Features and Stability
Conventional HB	Magnetite-derived Fe (Fe_3_O_4_) reduced in situ to α-Fe, promoted with K_2_O, Al_2_O_3_, CaO/SiO_2_	400–500; 150–250; 10^4^–10^5^	5–50	100–200	Multi-bed adiabatic reactors with inter-stage cooling and high gas hourly space velocities; single-pass NH_3_ typically 10–20 mol% with high overall yield via recycle. Catalysts are extremely robust, with effective lifetimes of several years between regenerations under carefully controlled feed purity and operating conditions.
Ru-based high-pressure HB	Promoted Ru on carbon or Ru on alkaline earth oxides (e.g., Ru/C, Ru/graphite, Ru/Ba–C)	380–450; 80–120; 10^4^–10^5^	20–100	80–100	Fixed-bed reactors operated at somewhat lower pressures than Fe for comparable outlet NH_3_ due to higher intrinsic Ru activity; loop design and space velocities similar to Fe plants. Long-term stability can be excellent when using optimized supports and promoters, but Ru cost and potential issues such as carbon methanation or support restructuring constrain widespread deployment and require stringent control of operating conditions.

## 5. Experimental Evidence for Associative Pathways on Single-Atom and Atomic-Cluster Catalysts

Although associative pathways are frequently invoked for SAC and SAA catalysts, rigorous experimental confirmation is still limited, and for many systems, the proposed mechanisms rely heavily on theoretical profiles complemented by indirect kinetic signatures rather than direct detection of all surface intermediates. This limited mechanistic resolution is itself a key challenge for SAC development, as it complicates the translation of DFT-derived descriptors into robust design rules without further operando validation. In this section, we restrict the term “associative” to systems that satisfy at least one of the following conditions: (i) apparent activation energies significantly below the 80–140 kJ mol^−1^ range typically associated with N_2_-dissociation-limited Haber–Bosch catalysts, together with clear kinetic changes when going from nanoparticles to single atoms or very small clusters; (ii) direct or quasi-direct detection of hydrogenated N_2_ intermediates (e.g., N_2_H, NNH_x_) by temperature-programmed desorption mass spectrometry (TPD–MS) or in situ spectroscopy techniques; and (iii) microkinetic/DFT studies on realistic SAC and SAA models that show dissociative N_2_ splitting to be both thermodynamically and kinetically disfavored compared to N_2_ hydrogenation, with *NH_x_ formation as the rate-determining step [17,25,37].

One of the clearest thermocatalytic examples is the single-atom Fe catalyst FeHL–N–C reported by Jiang and co-workers, in which FeN_4_ sites are embedded in N-doped carbon and stabilized via a post-metal-replacement strategy starting from a porphyrinic MOF precursor [25]. Under mild conditions (300 °C, 0.1 MPa), FeHL–N–C reached NH_3_ formation rates of 558 µmol g^−1^ h^−1^, outperforming conventional fused Fe and Co_3_Mo_3_N catalysts under comparable or harsher conditions. Kinetic measurements revealed an apparent activation energy of 38.4 kJ mol^−1^ for FeHL–N–C, compared to 44.4 kJ mol^−1^ for a lower-loading single-atom Fe material (Fe20NC) and 80.2 kJ mol^−1^ for Fe80NC containing Fe nanoparticles. Interestingly, these low activation energy values were similar to those found for Co atomic clusters and Ru single-atom catalysts (≈38–55 kJ mol^−1^) that have been assigned associative pathways [17]. Most importantly, Ar-TPD–MS performed after NH_3_ synthesis over FeHL–N–C revealed desorption signals assigned to N_2_H species, formed by direct hydrogenation of N_2_ without prior N–N bond scission, which the authors interpreted as direct experimental evidence that N_2_ undergoes an associative mechanism in Fe single-atom sites, with *NH_x_ formation as the rate-determining step. Control experiments confirmed that the N-doped carbon support with K/Na promoter was inactive at 300 °C and 0.1 MPa, that Fe phthalocyanine with FeN_4_ sites but without carbon embedding is much less active, and that trace Zn in ZnvFe_1_–N–C does not influence the activity, thereby corroborating that the associative pathway is intrinsically linked to atomically dispersed Fe.

For Ru, several thermocatalytic single-atom systems show kinetic signatures and theoretical profiles consistent with associative N_2_ activation. Ru single atoms confined in pure siliceous S-1 zeolite (Ru SAs/S-1) consist of isolated four-coordinate Ru sites stabilized by the zeolite framework and Ba promotion, with no aggregation observed after extended operation at 633 K [24]. The Ba-promoted Ru SAs/S-1 catalyst (0.27 wt% Ru, 9 wt% Ba) was found to deliver very high NH_3_ synthesis rates and turnover frequencies in the 523–648 K range, clearly surpassing benchmark Cs–Ru/MgO, while exhibiting an apparent activation energy as low as 55 kJ mol^−1^, markedly lower than typical Ru nanoparticle catalysts. DFT calculations on a RuN_4_-like site embedded in S-1 revealed dissociative N_2_ adsorption to be strongly disfavored, with the most favorable pathway involving stepwise hydrogenation of end-on *N_2_ with late N–N bond cleavage, characteristic of a distal/alternating-type associative mechanism.

In a related system, Ru/CeO_2_ containing mainly sub-nanometric Ru nanoclusters with a significant fraction of highly dispersed Ru sites exhibits the highest NH_3_ synthesis rate at 350–450 °C and 10–30 bar, with kinetic analysis showing an apparent activation energy of 64 kJ mol^−1^ and the absence of H_2_ poisoning [28], behavior that cannot be rationalized by a simple N_2_-dissociation-limited mechanism on extended Ru surfaces. DFT calculations on Ru–CeO_2_ models revealed that, for highly dispersed Ru species, N_2_ dissociation is less favorable than N_2_ hydrogenation and that mixed dissociative/associative routes involving hydrogenated N_2_ intermediates provide lower energy pathways to NH_3_. Although N_2_H-type intermediates were not directly observed in these Ru-based catalysts, the combination of low apparent activation energies, pronounced size effects upon going from nanoparticles to highly dispersed Ru species, and theoretical energetics that hinders pure dissociative N_2_ splitting provided consistent evidence that associative (or mixed associative–dissociative) mechanisms become operative under mild thermocatalytic conditions.

Beyond Fe and Ru, several Co-based single-atom and nanostructured catalysts have also shown features usually associated with associative N_2_ activation. For example, a Co–N–C single-atom catalyst exhibits an apparent activation energy of 50 ± 7 kJ mol^−1^, no detectable methanation of the carbon support, and Ar-TPD–MS signals dominated by N_2_H and N_2_H_2_ rather than NH/NH_2_, indicating preferential hydrogenation of N_2_ without direct N≡N dissociation and thereby suggesting a different N_2_ activation regime on these Co single-atom sites [39]. For Co_2_ atomic clusters (Co_2_ ACCs) on N-doped carbon, Arrhenius analysis between 325 and 380 °C at 1 MPa revealed an apparent activation energy as low as 49 kJ mol^−1^, much lower than the value measured for Co nanoparticles (75 kJ mol^−1^). Kinetic tests revealed that NH_3_ rates increased with pressure up to 3 MPa and that CH_4_ formation remained negligibly low over 24 h at 400 °C and 1 MPa, indicating the absence of strong H_2_ poisoning and methanation and thus a different activation regime than classical Co or Ru/C catalysts. Interestingly, in situ Co K-edge XANES/EXAFS and quasi-in situ N K-edge NEXAFS revealed strong metal–support and inter-cluster interactions with electron transfer from Co d orbitals to N_2_ π* orbitals and the build-up of N-containing surface intermediates consistent with NNH_x_ species rather than only atomic N. Complementary in situ DRIFTS under 25% N_2_/75% D_2_ at 400 °C detected bands assignable to hydrogenated N_2_ intermediates (NNH_x_/N_2_H_2_), and, together with the low activation energy, led to the authors to conclude that on Co_2_ ACCs, N_2_ was directly hydrogenated to NNH_x_ so that the N≡N bond escaped direct dissociation [40].

DFT studies have also supported the idea that small clusters and SACs can favor associative N_2_ hydrogenation via NNH_x_ intermediates. In an interesting study, Liu et al. [42] studied Fe_3_ sites supported on θ-Al_2_O_3_(010) and showed that N_2_ activation proceeds most favorably via an associative NNH pathway rather than direct dissociation. First, N_2_ adsorbs in a configuration on the Fe_3_ cluster such that substantial back-donation takes place (Bader charge: −1.13 e^-^; N–N elongated from 1.10 to 1.26 Å). For this configuration, the barrier for direct N_2_ dissociation remained high (1.89 eV), whereas hydrogenation to NNH required only 0.98 eV, with H_2_ dissociation itself being essentially barrierless (0.05 eV) once formed. Interestingly, microkinetic modeling revealed that, at 700 K and 100 bar, the associative mechanism contribution to the TOF was six orders of magnitude larger than the dissociative channel, yielding an overall TOF comparable to a Ru B_5_ site. These results are, however, predictive in nature and still await systematic experimental validation under HB conditions. For Co–N–C single-atom catalysts, Wang et al. [39] found that direct N_2_ dissociation on an isolated Co site was kinetically prohibitive, while hydrogenation of adsorbed N_2_ on Co_1_–N_3.5_ to yield HNNH, NH–NH_3_ and NH_2_–NH_4_ was energetically accessible; breaking the NH_2_–NH_4_ bond to release two NH_3_ molecules was identified as the key barrier, consistent with an Eley–Rideal-type associative route in which intact N_2_ is hydrogenated before N–N cleavage. Finally, Ren et al. [37] showed, for Ru_1_–Mo_2_CO_x_ SAC on defective Mo_2_CO_2_ MXene, that N_2_ adsorption is strongest in a side-on configuration involving Ru and neighboring Mo atoms, with 0.87 e charge transfer and elongation of the N–N bond to 1.24 Å, allowing the lowest-energy channel to be an associative pathway I (Figure 1) where N_2_ is first hydrogenated to NNH_2_, after which the N–N cleavage from NNH_2_ has a low barrier of only 0.50 eV, and the overall rate-determining step is the final hydrogenation from NH_2_ to NH_3_ (1.26 eV), whereas direct N_2_ dissociation requires 1.56 eV and is thus disfavored under mild conditions [31].

## 6. Outlook and Future Directions

Recent advances reviewed here show that SACs and sub-nanometric clusters can, in some cases, approach competitive NH_3_ productivities at 200–400 °C and ≤30 bar, especially when Ru and Fe single sites are embedded in zeolites, reducible oxides, N-doped carbons, or MOF-derived carbon matrices. However, the number of systems that truly combine high site density, low apparent activation energies, and multi-hundred-hour stability remains limited, and most studies still rely on carefully tuned laboratory conditions. More broadly, several cross-cutting challenges must be overcome before SAC-type catalysts can be considered realistic alternatives to state-of-the-art Fe and Ru formulations. These include the difficulty of synthesizing high-loading, structurally well-defined single-site materials at scale; potential deactivation via atom migration, support reconstruction, or carbon methanation during prolonged operation; the scarcity and cost of Ru for large-scale deployment; and the current scarcity of long-term tests under industrial space velocities, dynamic green-H_2_ operation, and realistic impurity levels. Addressing these limitations will be as important as further improving intrinsic activity.

A key priority is to generalize the synthetic methodologies that underpin the most successful systems (e.g., MOF-derived single-site frameworks, vacancy-templated post-metal replacement, and cluster-sized ensembles such as Co_2_) into scalable routes that provide precise control over metal loading, coordination, and proximity between distinct active motifs. Particular emphasis should be placed on non-noble catalysts since earth-abundant sites can realistically rival Ru when their coordination environment and electronic structure are adequately engineered. On the mechanistic side, systematic kinetic studies and operando spectroscopy are needed to quantify how associative versus dissociative pathways evolve with the number of metal sites and support polarity, and to link reaction orders and apparent activation energies to specific N_2_H_x_ intermediates across different material families, particularly for those SAC and cluster systems where the current mechanistic picture is predominantly based on DFT.

In addition to their excellent intrinsic activity, Ru-based single-atom and cluster catalysts face non-trivial economic and supply constraints that limit their scalability for global ammonia production. Ru is a noble metal with relatively low crustal abundance and a highly concentrated mining and refining infrastructure, which translates into high and volatile prices, as well as potential supply risk under rapid demand growth. While single-atom and sub-nanometric designs substantially reduce the Ru loading required per unit NH_3_ produced, they do not eliminate the underlying scarcity and cost issues, especially if such catalysts were to be deployed at the scale of the existing Haber–Bosch industry. These considerations strengthen the case for using Ru-based systems primarily as mechanistic benchmarks and proof-of-concept platforms for associative N_2_ activation under mild conditions, while prioritizing the parallel development of earth-abundant Fe-, Co- and Mo-based single-atom and cluster catalysts as more sustainable long-term solutions.

While this aspect is typically overlooked, the performance metrics of SAC-type catalysts must increasingly be evaluated under dynamic, electrified Haber–Bosch operation rather than under steady, idealized lab conditions. In practice, coupling to intermittent green-H_2_ supplies will impose frequent stop–start cycles, ramping in temperature and pressure, and large swings in H_2_/N_2_ ratio and space velocity, which can accelerate atom migration, support reconstruction, and promoter loss relative to conventional baseload operation. Under these conditions, additional constraints such as tolerance to water, oxygenated impurities, and trace CO/CO_2_ from upstream electrolysis become critical, since even ppm-level contaminants can induce site blocking or undesired nitride/carbon phases during repeated transients. SACs and sub-nanometric clusters are, in principle, well-positioned to meet several of these new requirements: their maximized metal utilization reduces the absolute demand for scarce elements, while strong metal–support/ligand coordination (e.g., Ru or Fe in zeolite cages, Fe–N_4_ motifs in conductive carbons, Co–N_x_ on hollow N-doped carbons) can suppress classical sintering pathways and mitigate H_2_ poisoning by tuning adsorption energetics. Consequently, future stability protocols for SACs should explicitly incorporate “electro-HB” stress scenarios, combining high-WHSV operation, periodic shutdown and restart, and controlled impurity dosing (H_2_O, O_2_, CO_x_, N_2_O, and sulfur traces) with multi-hundred-hour aging. Such tests must be accompanied by operando XAFS, environmental TEM, in situ vibrational spectroscopy, and isotopic labeling under transient, rather than only steady conditions, so that the evolution of single-atom environments, support defects, and promoter distributions can be followed in real time and quantitatively linked to losses in activity, selectivity, or H_2_-tolerance. Insights from these dynamic assays should then feed directly into the design of next-generation SAC architectures—for example, by identifying support chemistries and coordination motifs that retain isolated Fe, Co, or Ru centers and associative N_2_-activation pathways even under aggressive cycling with green-H_2_ feeds—thereby aligning SAC development with the actual demands of decentralized, low-carbon ammonia plants.

In parallel with advances in mechanistic understanding and synthesis, emerging data-driven approaches offer an additional route to accelerate the discovery of SACs and sub-nanometric clusters for thermocatalytic NH_3_ synthesis. Recent work has shown that DFT datasets for Ru and other transition-metal sites can be mined to build machine learning (ML) models that correlate local geometric and electronic descriptors (coordination number, d-band metrics, charge and the adsorption energies of N-intermediates) with computed activity and scaling relations for N_2_ activation and hydrogenation. Such surrogate models can then be used in high-throughput screening of candidate metal–support combinations (e.g., Ru/Fe/Co single atoms on oxides, carbons, MXenes) and to identify optimal windows of N and H binding energies where associative or weakened dissociative pathways become competitive, thereby narrowing the experimental search space for Haber–Bosch catalysts under mild conditions [17]. Importantly, ML approaches have begun to be applied to large in situ and operando XAFS datasets to automatically extract structural descriptors and correlate them with catalytic activity and deactivation, suggesting a powerful route to quantify how single-atom environments evolve under realistic reaction conditions [43]. Embedding such data-driven stability metrics into screening and design pipelines could become essential for prioritizing SACs and clusters that are not only highly active but also intrinsically robust over industrial timescales, including under Haber–Bosch-like cycling [43].

Finally, although several Ru-, Fe- and Co-based SACs and sub-nanometric clusters now show promising 20–150 h stability under Haber–Bosch-like conditions, current evidence is still largely based on ex situ or post-mortem characterization, offering only a partial view of deactivation phenomena such as atom migration, support reconstruction, poisoning or phase transformations. To translate these model systems into practical NH_3_ synthesis catalysts, future work should systematically combine long-term accelerated aging protocols (e.g., cycling in T, p and H_2_/N_2_ ratio; exposure to realistic feed impurities) with operando XAFS, environmental TEM, in situ vibrational spectroscopy and isotopic labeling, so that dynamic restructuring of single sites and supports can be directly correlated with activity and selectivity changes and, ultimately, used to design SAC architectures that are intrinsically resistant to sintering, poisoning and carbon/nitride formation under industrially relevant conditions.

Beyond thermocatalytic routes, electrocatalytic nitrogen reduction (eNRR) has emerged as a complementary strategy for ammonia production under ambient conditions [44]. While this approach relies on fundamentally different descriptors and performance metrics (e.g., Faradaic efficiency, current density, and electrolyte effects), it shares a common interest in atomically precise active sites with thermocatalysis. In this context, single-atom catalysts have been extensively explored in eNRR systems as a means to control N_2_ adsorption and suppress competing hydrogen evolution [45]. Looking forward, bridging the gap between thermocatalytic and electrocatalytic ammonia synthesis may open new opportunities for catalyst design, particularly by leveraging insights from coordination chemistry and structure-activity relationships across both fields. Such cross-fertilization could contribute to the development of more efficient and scalable routes for sustainable ammonia production.

## 7. Conclusions

This review has examined how downsizing classical Fe and Ru catalysts to SACs and sub-nanometric clusters can be used to reshape the mechanistic limitations of thermocatalytic ammonia synthesis at mild conditions. Across a broad set of systems, a consistent picture emerges in which isolated sites promote associative N_2_ activation, which can shift the rate-determining step away from direct N≡N cleavage toward N_2_H_x_ hydrogenation and, in many reported systems, reduce apparent activation energies and alleviate hydrogen poisoning relative to classical Fe and Ru catalysts, although so far mainly under laboratory conditions. Ru-based SACs embedded in zeolites, reducible oxides such as CeO_2_, graphitized carbons, and MXenes demonstrate that strong and often bifunctional metal–support interactions can simultaneously stabilize cationic single atoms, supply electrons and spillover hydrogen, and, in tandem configurations, couple associative and dissociative pathways to reach high NH_3_ productivities with long-term stability.

The rapidly expanding family of non-noble SACs and clusters suggests that earth-abundant metals may, in favorable cases, challenge Ru performance when their coordination environments and number of sites are precisely engineered. High-loading Fe–N_4_ catalysts obtained via MOF-derived post-metal-replacement strategies exemplify how vacancy-templated routes overcome the traditional trade-off between single-site stabilization and metal loading, delivering activities at 300 °C and 1 bar that surpass fused-iron and even Ru benchmarks. Complementary studies on Co single atoms and Co_2_ clusters supported on N-doped carbons show that tuning the Co–N coordination number and introducing well-defined two-atom ensembles further lowers activation barriers, yields favorable N_2_ and H_2_ reaction orders, and maintains structural integrity over tens of hours on stream with negligible methanation. Despite this progress, most reported SAC systems have been evaluated under relatively idealized laboratory conditions and over modest times on stream. Future work must therefore focus on translating the most promising design principles into scalable syntheses while enabling operation at industrially relevant space velocities. Furthermore, very few non-noble SACs have been tested beyond 50–150 h, so long-term stability, resistance to feed impurities, and behavior under load cycling remain largely open questions.

## Data Availability

No new data were created.

## References

[1] Smith C., Hill A.K., Torrente-Murciano L. (2020). Current and Future Role of Haber–Bosch Ammonia in a Carbon-Free Energy Landscape. Energy Environ. Sci..

[2] Jeerh G., Zhang M., Tao S. (2021). Recent Progress in Ammonia Fuel Cells and Their Potential Applications. J. Mater. Chem. A Mater..

[3] Zhai L., Liu S., Xiang Z. (2023). Ammonia as a Carbon-Free Hydrogen Carrier for Fuel Cells: A Perspective. Ind. Chem. Mater..

[4] Ravi M., Makepeace J.W. (2022). Facilitating Green Ammonia Manufacture under Milder Conditions: What Do Heterogeneous Catalyst Formulations Have to Offer?. Chem. Sci..

[5] Verschoor J.C., de Jongh P.E., Ngene P. (2024). Recent Advances in Thermocatalytic Ammonia Synthesis and Decomposition. Curr. Opin. Green Sustain. Chem..

[6] Ganti S.S.S., Roy P.K., Wagh N., Siva Sai K.N.S., Kumar S. (2025). Strategically Designed Catalysts for Ammonia Synthesis under Mild Conditions: Recent Advances and Challenges. J. Mater. Chem. A Mater..

[7] Jacobsen C.J.H., Dahl S., Clausen B.S., Bahn S., Logadottir A., Nørskov J.K. (2001). Catalyst Design by Interpolation in the Periodic Table: Bimetallic Ammonia Synthesis Catalysts. J. Am. Chem. Soc..

[8] Varadwaj P.R., Marques H.M., Grabowski I. (2025). Ammonia Synthesis over Transition Metal Catalysts: Reaction Mechanisms, Rate-Determining Steps, and Challenges. Int. J. Mol. Sci..

[9] Wang S., Petzold V., Tripkovic V., Kleis J., Howalt J.G., Skúlason E., Fernández E.M., Hvolbæk B., Jones G., Toftelund A. (2011). Universal Transition State Scaling Relations for (de)Hydrogenation over Transition Metals. Phys. Chem. Chem. Phys..

[10] Abild-Pedersen F., Greeley J., Studt F., Rossmeisl J., Munter T.R., Moses P.G., Skúlason E., Bligaard T., Nørskov J.K. (2007). Scaling Properties of Adsorption Energies for Hydrogen-Containing Molecules on Transition-Metal Surfaces. Phys. Rev. Lett..

[11] Hattori M., Iijima S., Nakao T., Hosono H., Hara M. (2020). Solid Solution for Catalytic Ammonia Synthesis from Nitrogen and Hydrogen Gases at 50 °C. Nat. Commun..

[12] Marakatti V.S., Gaigneaux E.M. (2020). Recent Advances in Heterogeneous Catalysis for Ammonia Synthesis. ChemCatChem.

[13] Peng X., Zhang M., Zhang T., Zhou Y., Ni J., Wang X., Jiang L. (2024). Single-Atom and Cluster Catalysts for Thermocatalytic Ammonia Synthesis at Mild Conditions. Chem. Sci..

[14] Long J., Fu X., Xiao J. (2020). The Rational Design of Single-Atom Catalysts for Electrochemical Ammonia Synthesis: Via a Descriptor-Based Approach. J. Mater. Chem. A Mater..

[15] Zhang Y., Li S., Sun C., Wang P., Yang Y., Yi D., Wang X., Yao J. (2022). Understanding and Modifying the Scaling Relations for Ammonia Synthesis on Dilute Metal Alloys: From Single-Atom Alloys to Dimer Alloys. ACS Catal..

[16] Shen F., He S., Tang X., Liu Y., Wang Y., Yin Y., Lv X., Fu W., Zou Y., Jiang G. (2025). Breaking Linear Scaling Relation Limitations on a Dual-Driven Single-Atom Copper-Tungsten Oxide Catalyst for Ammonia Synthesis. Angew. Chem. Int. Ed..

[17] Ghoreishian S.M., Shariati K., Huh Y.S., Lauterbach J. (2023). Recent Advances in Ammonia Synthesis over Ruthenium Single-Atom-Embedded Catalysts: A Focused Review. Chem. Eng. J..

[18] Kilic M.E., Jena P. (2025). Single-Atom vs. Single-Superatom as Catalysts for Ammonia Production. Chem. Commun..

[19] Zhao Y., Xu J., Huang Z., Wang Y., Li Y., Wei H. (2025). Tuning Metal-Phthalocyanine 2D Covalent Organic Frameworks for the Nitrogen Reduction Reaction: Cooperativity of Transition Metals and Organic Linkers. ACS Appl. Mater. Interfaces.

[20] Ou L., Jin J., Chen Y. (2021). Theoretical Insights into the Electroreduction Mechanism of N_2_ to NH_3_ from an Improved Au(111)/H_2_O Interface Model. RSC Adv..

[21] Chalkley M.J., Drover M.W., Peters J.C. (2020). Catalytic N_2_-to-NH_3_ (or -N_2_H_4_) Conversion by Well-Defined Molecular Coordination Complexes. Chem. Rev..

[22] Zhang Y., Li J., Zhou Y., Au C., Wang X., Jiang L. (2025). Recent Progress of Thermocatalytic Ammonia Synthesis via an Associative Mechanism. Fundam. Res..

[23] Yue Y., Chen Y., Zhang X., Qin J., Zhang X., Liu R. (2022). High-Throughput Screening of Highly Active and Selective Single-Atom Catalysts for Ammonia Synthesis on WB2 (0 0 1) Surface. Appl. Surf. Sci..

[24] Qiu J.Z., Hu J., Lan J., Wang L.F., Fu G., Xiao R., Ge B., Jiang J. (2019). Pure Siliceous Zeolite-Supported Ru Single-Atom Active Sites for Ammonia Synthesis. Chem. Mater..

[25] Jiang Y., Chen Z., Peng T., Jiao L., Pan X., Jiang H.L., Bao X. (2025). Single-Atom Fe Catalysts With Improved Metal Loading for Efficient Ammonia Synthesis Under Mild Conditions. Angew. Chem. Int. Ed..

[26] Ye T.N., Lu Y., Kobayashi Y., Li J., Park S.W., Sasase M., Kitano M., Hosono H. (2020). Efficient Ammonia Synthesis over Phase-Separated Nickel-Based Intermetallic Catalysts. J. Phys. Chem. C.

[27] Arroyo-Caire J., Jiang Y., Diaz-Perez M.A., Lara-Angulo M.A., Miyazaki M., Serrano-Ruiz J.C., Kitano M., Hosono H. (2023). CeNix Alloys as Catalysts for Ammonia Synthesis: Insights on Ni–CeN Surface Layer Formation and Its Impact. ACS Catal..

[28] Sivan S.E., Kang K.H., Han S.J., Francis Ngome Okello O., Choi S.Y., Sudheeshkumar V., Scott R.W.J., Chae H.J., Park S., Lee U.H. (2022). Facile MOF-Derived One-Pot Synthetic Approach toward Ru Single Atoms, Nanoclusters, and Nanoparticles Dispersed on CeO_2_ Supports for Enhanced Ammonia Synthesis. J. Catal..

[29] Zhou Y., Yang B., Wang L., Su K., Liu B., Fang H., Yue K., Wang X., Qi H., Zheng L. (2025). Ru Single Atom and Nanoparticle Tandem Catalyst Unlocking High-Efficiency Ammonia Synthesis under Mild Conditions. J. Am. Chem. Soc..

[30] Wang X., Li L., Fang Z., Zhang Y., Ni J., Lin B., Zheng L., Au C., Jiang L. (2020). Atomically Dispersed Ru Catalyst for Low-Temperature Nitrogen Activation to Ammonia via an Associative Mechanism. ACS Catal..

[31] Zhang Y., Zhang M., Zhou Y., Yang L., Lin B., Ni J., Zheng L., Wang X., Au C.T., Jiang L. (2022). Insight into the Critical Role of Strong Interaction between Ru and Co in RuCo Single-Atom Alloy Structure for Significant Enhancement of Ammonia Synthesis Performance. J. Catal..

[32] Gawande M.B., Fornasiero P., Zbořil R. (2020). Carbon-Based Single-Atom Catalysts for Advanced Applications. ACS Catal..

[33] Arroyo-Caire J., Duran-Uribe E.S., Lara-Angulo M.A., Diaz-Perez M.A., Sepúlveda-Escribano A., Serrano-Ruiz J.C. (2025). Efficient CeO_2_ and CeO_2_—Al_2_O_3_ Supports for Ru as 3rd Generation Ammonia Synthesis Catalysts: Enhanced Kinetic Mechanism over Commercial Ru/CeO_2_. Catal. Sci. Technol..

[34] Lin B., Fang B., Wu Y., Li C., Ni J., Wang X., Lin J., Au C., Jiang L. (2021). Enhanced Ammonia Synthesis Activity of Ceria-Supported Ruthenium Catalysts Induced by CO Activation. ACS Catal..

[35] Li W., Liu P., Niu R., Li J., Wang S. (2020). Influence of CeO_2_ Supports Prepared with Different Precipitants over Ru/CeO_2_ Catalysts for Ammonia Synthesis. Solid State Sci..

[36] Singh S., Komarala E.P., Kim S.J., Yavuz C.T., Maghrabi L.M., Singh N., Harfouche M., Sabastian V., Malina O., Bakandritsos A. (2025). Robust Ru Single-Atom Alloy Catalysts Coupled with Adjacent Fe-Site for Highly Stable Ammonia Synthesis under Mild Conditions. Appl. Surf. Sci..

[37] Ren Y., Zhang C., Wang H., Liang J.-X., Zhu C., Hu H.-S., Li J. (2025). Defective Ru1@Mo_2_CO Single-Atom Catalyst for Efficient Thermal Catalysis for Ammonia Synthesis. Chin. J. Struct. Chem..

[38] Chen Z., Ye Y., Peng T., Wu C., Li H., Pan X., Bao X. (2023). Iron-Single Sites Confined by Graphene Lattice for Ammonia Synthesis under Mild Conditions. ACS Catal..

[39] Wang X., Peng X., Chen W., Liu G., Zheng A., Zheng L., Ni J., Au C., Jiang L. (2020). Insight into Dynamic and Steady-State Active Sites for Nitrogen Activation to Ammonia by Cobalt-Based Catalyst. Nat. Commun..

[40] Peng X., Cai H., Zhou Y., Ni J., Wang X., Lin B., Lin J., Zheng L., Au C., Jiang L. (2022). Studies of a Highly Active Cobalt Atomic Cluster Catalyst for Ammonia Synthesis. ACS Sustain. Chem. Eng..

[41] Zhou Y., Wang C., Peng X., Zhang T., Wang X., Jiang Y., Qi H., Zheng L., Lin J., Jiang L. (2022). Boosting Efficient Ammonia Synthesis over Atomically Dispersed Co-Based Catalyst via the Modulation of Geometric and Electronic Structures. CCS Chem..

[42] Liu J.-C., Ma X.-L., Li Y., Wang Y.-G., Xiao H., Li J. (2018). Heterogeneous Fe_3_ Single-Cluster Catalyst for Ammonia Synthesis via an Associative Mechanism. Nat. Commun..

[43] Timoshenko J., Frenkel A.I. (2019). “Inverting” X-Ray Absorption Spectra of Catalysts by Machine Learning in Search for Activity Descriptors. ACS Catal..

[44] Ghoshal S., Ghosh A., Roy P., Ball B., Pramanik A., Sarkar P. (2022). Recent Progress in Computational Design of Single-Atom/Cluster Catalysts for Electrochemical and Solar-Driven N_2_ Fixation. ACS Catal..

[45] Choi C., Back S., Kim N.-Y., Lim J., Kim Y.-H., Jung Y. (2018). Suppression of Hydrogen Evolution Reaction in Electrochemical N_2_ Reduction Using Single-Atom Catalysts: A Computational Guideline. ACS Catal..

